# Nutritional risk screening score as an independent predictor of nonventilator hospital-acquired pneumonia: a cohort study of 67,280 patients

**DOI:** 10.1186/s12879-021-06014-w

**Published:** 2021-04-01

**Authors:** Zhihui Chen, Hongmei Wu, Jiehong Jiang, Kun Xu, Shengchun Gao, Le Chen, Haihong Wang, Xiuyang Li

**Affiliations:** 1 Department of Epidemiology and Biostatistics, and Centre for Clinical Big Data Statistics, Second Affiliated Hospital, Zhejiang University College of Medicine, 866 Yuhangtang Road, Hangzhou, 310058 China; 2 Department of Infection Control, Wenzhou people’s Hospital, Wenzhou, China; 3 XingLin Information Technology Company, Hangzhou, China

**Keywords:** Malnutrition, Screening, Hospital-acquired pneumonia, Aspiration pneumonia, Cohort study

## Abstract

**Background:**

Currently, the association of nutritional risk screening score with the development of nonventilator hospital-acquired pneumonia (NV-HAP) is unknown. This study investigated whether nutritional risk screening score is an independent predictor of NV-HAP.

**Methods:**

This retrospective cohort study was conducted between September 2017 and June 2020 in a tertiary hospital in China. The tool of Nutritional Risk Screening 2002 (NRS-2002) was used for nutritional risk screening. A total score of ≥3 indicated a patient was “at nutritional risk.” Logistic regression was applied to explore the association between the NRS score and NV-HAP.

**Results:**

A total of 67,280 unique patients were included in the study. The incidence of NV-HAP in the cohort for the NRS < 3 and ≥ 3 NRS group was 0.4% (232/62702) and 2.6% (121/4578), respectively. In a multivariable logistic regression model adjusted for all of the covariates, per 1-point increase in the NRS score was associated with a 30% higher risk of NV-HAP (OR = 1.30; 95%CI:1.19–1.43). Similarly, patients with NRS score ≥ 3 had a higher risk of NV-HAP with an odds ratio (OR) of 2.06 (confidence interval (CI): 1.58–2.70) than those with NRS score < 3. Subgroup analyses indicated that the association between the NRS score and the risk of NV-HAP was similar for most strata. Furthermore, the interaction analyses revealed no interactive role in the association between NRS score and NV-HAP.

**Conclusion:**

NRS score is an independent predictor of NV-HAP, irrespective of the patient’s characteristics. NRS-2002 has the potential as a convenient tool for risk stratification of adult hospitalized patients with different NV-HAP risks.

**Supplementary Information:**

The online version contains supplementary material available at 10.1186/s12879-021-06014-w.

## Introduction

Hospital-acquired pneumonia (HAP) is one of the most frequent types of healthcare-associated infections (HAIs) [1]. It includes two distinct subgroups: nonventilator hospital-acquired pneumonia (NV-HAP) and ventilator-associated pneumonia (VAP). Currently, more than two-thirds of HAP cases are of the NV-HAP type [2, 3]. Although both NV-HAP and VAP impose enormous clinical and economic burdens clinical and economic burdens [4–6], evidence suggests that NV-HAP has higher overall medical costs and greater overall mortality than VAP [6]. However, literature concerning NV-HAP is rare. Most studies and prevention strategies targeting HAP have primarily focused on VAP [2]. Studies have revealed that modifiable risk factors, such as swallowing evaluation and oral care, can reduce the risk of NV-HAP [7, 8]. Therefore, the search for additional modifiable risk factors of NV-HAP is urgently needed.

Factors thought to be influencing NV-HAP have been explored in several studies [9, 10], were most patient-related risk factors associated with an increased NV-HAP morbidity cannot be corrected [7, 11]. Malnutrition, as an important risk factor for HAIs [12], is highly prevalent in hospitalized adult patients. The prevalence of malnutrition ranges from 20 to 50% in hospitalized patients [13]. With appropriate nutritional support therapy, malnutrition is potentially reversible. The nutritional support therapy is therefore becoming an appealing target for prevention and management of HAIs, including the NV-HAP [14]. To identify important nutritional targets, the association between nutritional risk and NV-HAP should be explored.

The NRS-2002 is a validated tool for nutritional screening of patients between 18 to 90 years of age who have or are at risk of malnutrition. The tool includes standard screening parameters, such as body mass index (BMI), patient’s age, weight loss, dietary intake, and severity of underlying disease [15]. The NRS-2002 score ranges from 0 to 7, and a total score of ≥3 indicates that a patient is “at nutritional risk”. This tool has been confirmed and validated by several studies worldwide and is widely used for screening hospitalized patients who are nutritionally at risk [16–18]. Several studies have identified the nutritional risk screening (NRS) score as an independent predictor of HAIs [12], such as surgical site infections [19]. However, no longitudinal data concerning the the association of NRS score with the risk of NV-HAP.

Thus, we investigated the relationship between nutritional risk screening scores and NV-HAP.P.

## Methods

### Data sources and study population

We conducted a retrospective cohort study, including all inpatients admitted between September 1, 2017, through June 30, 2020, at Wenzhou People’s Hospital (a 1500-bed tertiary teaching hospital in Zhejiang, China). Patients who were pregnant, younger than 18 years of age or greater than 90 years of age, length of hospital stay < 48 h, received mechanical ventilation during hospitalization, and lack of nutritional risk screening score were excluded from the analysis. If patients were readmitted during the study period, only their first admission was considered. The study was approved by the Ethics Review Committee of Wenzhou people’s Hospital [approval no. WRY2018070]. Given the retrospective nature of the study, the requirement of informed consent was waived. This paper was reported in line with the STROBE guidelines [20].

### Nutritional risk screening (NRS)

All adult patients, except pregnant women, underwent nutritional risk screening. The nutritional risk screening was performed within 24 h after admission by ward nursing staff who were trained to conduct using the NRS-2002 tool.

### Outcome

The NV-HAP data was obtained from the Xinglin system [21]. This system is a web-based, real-time monitoring system of nosocomial infection, which automatically identify symptoms of infections and clinical data such as fever, positive bacterial culture, and elevated inflammatory response markers for initial diagnoses. Meanwhile, the system is also used to transfer nosocomial infection cases identified by the clinicians to senior infection control practitioner for a definitive diagnosis. In case of a disagreement between the two sides, a consensus was made via discussions. Nonventilator hospital-acquired pneumonia (NV-HAP) is defined as a pneumonia not present or incubating at the time of hospital admission and occurring at least 48 h after admission in patients not receiving invasive mechanical ventilation during hospitalization [22]. The diagnostic criteria used in the present study for NV-HAP strictly adhered to the 2018 version of the Chinese guidelines [22]. The 2018 version of the Chinese guidelines is compatible with the guidelines issued by the American Thoracic Society [23].

### Covariates

Admission data collected from the electronic medical record system included age; sex; drinking status; smoking status, comorbidities, admission category, Barthel Index, Morse Fall Scale, and season of admission. The Barthel Index (BI) [24] and the Morse Fall Scale [25] were used to assess the patient’s level of independence and nursing-related complications, respectively. Charlson comorbidity index (CCI) was used to measure the burden of comorbid conditions [26].

Based on the outcome and exposure to the hospital environment, we added a covariate termed “time at risk” into the model. For NV-HAP patients, “time at risk” was calculated as the number of days between the admission day and date of diagnosis of NV-HAP. For non-NV-HAP patients, the “time at risk” corresponded to the total hospital days. We collected information from the Xinglin system concerning clinical procedures (including a central venous catheter, indwelling urinary catheter, surgery, parenteral nutrition, and enteral tube feeding), other nosocomial infections, and the use of specific classes of medications such as antacids, sedatives, nonsteroidal anti-inflammatory drug (NSAID), systemic steroid, inhaled steroid, and anticoagulant during the “time at risk” period.

### Statistical analysis

Non-normal continuous variables were presented as medians (Q1-Q3) and compared using Mann-Whitney U test. Categorical variables were presented as numbers (proportion) and compared using Chi-square test or Fisher’s exact test. To avoid bias caused by missing NRS score data, the characteristics of individuals with missing data were compared with those with complete data. As less than 1% of the covariates were missing, the missing data were not dealt with. Logistic regression analyses were used to estimate odds ratios (ORs) and 95% confidence intervals (95% CIs) for the association between NRS score and risk of NV-HAP. Firstly, possible collinearity was assessed based on the variance inflation factor (VIF); variables with VIF > 10 were removed from the Model. Secondly, we used four different logistic regression models to examine the associations of nutritional risk screening score and the risk for NV-HAP. The Non-adjusted Model examined the association between NRS score and NV-HAP without adjustment for any covariates. Model I included demographic characteristics (age and sex). Model II made an additional adjustment for variables that, when added to this model, changed in effect estimate of more than 10% [27], included the covariates in Model I plus stroke, Charlson comorbidity index, time of risk, central venous catheter, enteral tube feeding, Barthel Index, Morse Fall Scale. The association of each covariate with NV-HAP is shown in Supplementary Table S2. Model III (the fully adjusted model) included the covariates in Model II plus the other covariates listed in Table 1. Thirdly, these analyses were performed on unique patients, making it possible for a patient with multiple admissions; therefore, risk estimation was also performed using the generalized estimation equation (GEE) method with a logit link and exchangeable correlation matrix while adjusting for the possible dependence in the outcome introduced by repeated admissions. Finally, to assess the homogeneity of effects, subgroup analyses and interaction tests were performed for the covariates shown in Table 1.

**Table 1:** Baseline characteristics of the study population

Demographics	Total (*n* = 67,280)	NRS score < 3 (*n* = 62,702)	NRS score ≥ 3 (*n* = 4578)	*P* value
Age (years), median (Q1-Q3)	51 (37–65)	50 (37–64)	68 (43–78)	< 0.001
Male, n (%)	28,684 (42.6)	26,499 (42.3)	2185 (47.7)	< 0.001
Drinking status, n (%)				< 0.001
Never drinker	57,117 (84.9)	53,246 (84.9)	3871 (84.5)	
Current drinker	7802 (11.6)	7346 (11.7)	456 (10.0)	
Former drinker	2119 (3.1)	1890 (3.0)	229 (5.0)	
Missing	242(0.4)	220 (0.4)	22 (0.5)	
Smoking status, n (%)				< 0.001
never smoker	55,266 (82.1)	51,584 (82.3)	3682 (80.4)	
Current smoker	8634 (12.9)	8092 (12.9)	542 (11.8)	
Former smoker	3230 (4.8)	2886 (4.6)	344 (7.6)	
Missing	150(0.2)	140 (0.2)	10 (0.2)	
Comorbidities, n (%)				
COPD	803 (1.2)	651 (1.0)	152 (3.3)	< 0.001
Swallow disability	126 (0.2)	63 (0.1)	63 (1.4)	< 0.001
Stroke	7237 (10.8)	6072 (9.7)	1165 (25.4)	< 0.001
Diabetes mellitus	9612 (14.3)	8805 (14.0)	807 (17.6)	< 0.001
Peptic ulcer disease	2236 (3.3)	2128 (3.4)	108 (2.4)	< 0.001
Moderate or severe renal disease	2944 (4.4)	2733 (4.4)	211 (4.6)	0.424
Liver disease	11,993 (17.8)	11,560 (18.4)	433 (9.5)	< 0.001
Congestive heart failure	328 (0.5)	284 (0.5)	44 (1.0)	< 0.001
Solid tumour	4962 (7.4)	4272 (6.8)	690 (15.1)	< 0.001
CCI (points), median (Q1- Q3)	1 (0–3)	1 (0–3)	4 (1–5)	< 0.001
Time of risk (days), median (Q1- Q3)	7 (4–10)	7 (4–10)	10 (6–16)	< 0.001
Admission category, n (%)				< 0.001
Internal medicine	27,769 (41.3)	25,487 (40.6)	2282 (49.8)	
Surgery	19,556 (29.1)	18,110 (28.9)	1446 (31.6)	
Gynaecology	15,548 (23.1)	15,084 (24.1)	464 (10.1)	
Emergency department	3172 (4.7)	2902 (4.6)	270 (5.9)	
ICU	203 (0.3)	137 (0.2)	66 (1.4)	
Others	1032 (1.5)	982 (1.6)	50 (1.1)	
Clinical procedure, n (%)				
Central venous catheter	1762 (2.6)	1294 (2.1)	468 (10.2)	< 0.001
Indwelling urinary catheter	13,823 (20.5)	12,972 (20.7)	851 (18.6)	< 0.001
Surgery	20,979 (31.2)	20,161 (32.2)	818 (17.9)	< 0.001
Parenteral nutrition	1479 (2.2)	1077 (1.7)	402 (8.8)	< 0.001
Enteral tube feeding	5698 (8.5)	4849 (7.7)	849 (18.5)	< 0.001
Barthel Index, n (%)				< 0.001
Independent	43,273 (64.3)	41,706 (66.5)	1567 (34.2)	
Slight dependency	6057 (9.0)	5601 (8.9)	456 (10.0)	
Moderate dependency	10,085 (15.0)	9109 (14.5)	976 (21.3)	
Severe dependency	6495 (9.7)	5461 (8.7)	1034 (22.6)	
Total dependency	1370 (2.0)	825 (1.3)	545 (11.9)	
Morse Fall Scale, n (%)				< 0.001
No Risk	41,008 (61.0)	39,220 (62.5)	1788 (39.1)	
Low Risk	21,697 (32.2)	19,805 (31.6)	1892 (41.3)	
High Risk	4575 (6.8)	3677 (5.9)	898 (19.6)	
Other nosocomial infections, n (%)	1137 (1.7)	983 (1.6)	154 (3.4)	< 0.001
Season of admission, n (%)				< 0.001
Spring	16,431 (24.4)	15,356 (24.5)	1075 (23.5)	
Summer	18,652 (27.7)	17,451 (27.8)	1201 (26.2)	
Fall	14,497 (21.5)	13,390 (21.4)	1107 (24.2)	
Winter	17,700 (26.3)	16,505 (26.3)	1195 (26.1)	
In-hospital medications, n (%)				
Antacids	37,406 (55.6)	34,396 (54.9)	3010 (65.7)	< 0.001
Sedatives	6615 (9.8)	5889 (9.4)	726 (15.9)	< 0.001
NSAID	6503 (9.7)	5879 (9.4)	624 (13.6)	< 0.001
Systemic steroid	13,231 (19.7)	12,445 (19.8)	786 (17.2)	< 0.001
Inhaled steroid	4281 (6.4)	3760 (6.0)	521 (11.4)	< 0.001
Anticoagulant	8972 (13.3)	8229 (13.1)	743 (16.2)	< 0.001
NV-HAP	353(0.5)	232(0.4)	121(2.6)	< 0.001

Abbreviations: *NRS* Nutritional risk screening, *Q1* First quartile, *Q3* Third quartile, *COPD* Chronic obstructive pulmonary disease, *CCI* Charlson comorbidity index, *ICU* Intensive care unit, *NSAID* Nonsteroidal anti-inflammatory drug, *NV-HAP* Nonventilator hospital-acquired pneumonia

Statistical analyses were performed with the R software (version 3.4.3; http://www.R-project.org) and EmpowerStats software (www.empowerstats.com, X&Y solutions, Inc. Boston MA). A two-tailed *P*-value of ≤0.05 was considered to be statistically significant.

## Results

### Study participants and baseline characteristics

**Fig. 1:**
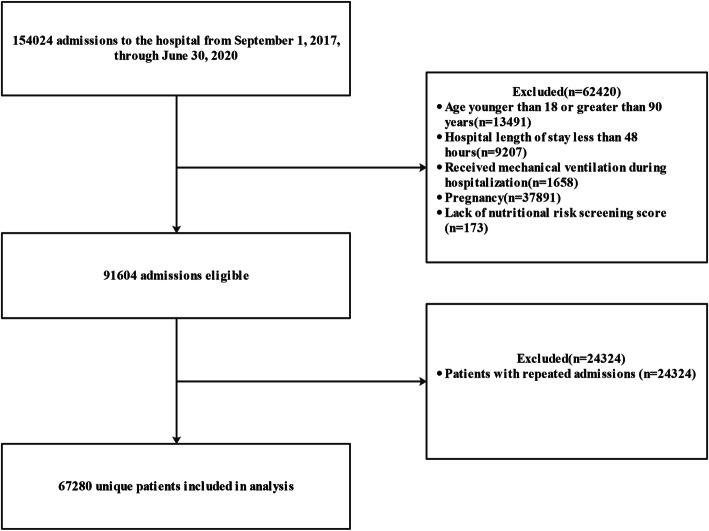
Flow chart of the study population

There were 154,024 admissions to the medical centre from September 1, 2017, through June 30, 2020. After excluding admissions with younger than 18 years of age or greater than 90 years of age (*n* = 13,491), length of hospital stay < 48 h (*n* = 9207), received mechanical ventilation during hospitalization (*n* = 1658), pregnancy (*n* = 37,891), lack of nutritional risk screening score (*n* = 173), and repeated admissions (*n* = 24,324), a total of 67,280 unique patients were included in the final analysis (Fig. 1). Baseline characteristics of study participants by NRS score are listed in Table 1. In the present study, 4578 (6.8%) patients were at nutritional risk (NRS-2002 ≥ 3). There were significant differences in baseline characteristics between patients with NRS score < 3 and those with NRS score ≥ 3 (Table 1).

### The incidence of NV-HAP according to NRS scores

**Fig. 2:**
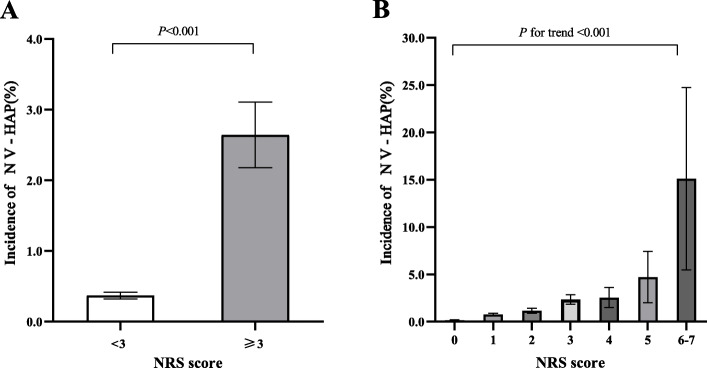
Incidence of NV-HAP among Chinese adults for the NRS < 3 group and NRS score ≥ 3 group (A), and for NRS score of 0, 1, 2, 3, 4,5, and ≥ 6, respectively (B). Abbreviations: NRS, nutritional risk screening; NV-HAP, nonventilator hospital-acquired pneumonia. Note: patients with NRS scores of 6 and 7 were combined for analysis due to small sample size; a total score of ≥3 indicated a patient is “at nutritional risk”

The incidence of NV-HAP in the cohort for the NRS < 3 group and NRS score ≥ 3 group was 0.4% (232/62702) and 2.6% (121/4578), respectively (Table 1). The proportion of patients with NV-HAP was significantly higher in the NRS ≥3 groups (Fig. 2a). The incidence of NV-HAP showed an NRS score-dependent increase (*P* for trend< 0.001). The incidence of NV-HAP was 0.2, 0.8, 1.1, 2.3, 2.5, 4.7, and 15.1% for NRS scores of 0, 1, 2, 3, 4,5, and ≥ 6, respectively (Fig. 2b).

### Relationship between NRS score and NV-HAP

Results of VIF analysis for variables showed that there was no collinearity bias (Table S1 in the Supplementary Appendix). The unadjusted and multivariate-adjusted analyses of the relationship between NRS score and NV-HAP are shown in Table 2. NRS score, whether considered a categorical or continuous variable, was independently associated with the risk of NV-HAP in different multivariate logistic regression models. Patients with NRS score ≥ 3 were at a higher risk of NV-HAP (OR = 7.31; 95%CI: 5.86, 9.31) than those with NRS score was < 3. After adjusting for age and sex (Model I), the association remained unchanged (OR = 4.39;95% CI: 3.47, 5.55).

**Table 2:** Association between nutritional risk screening score and nonventilator hospital-acquired pneumonia in multivariate logistic regression model

NRS score	Non-adjusted Model		Model I		Model II		Model III	
	OR (95% CI)	*P* value	OR (95% CI)	*P* value	OR (95% CI)	*P* value	OR (95% CI)	*P* value
Continuous, per 1-point increment	2.05(1.92–2.19)	< 0.001	1.74(1.62–1.87)	< 0.001	1.28 (1.18, 1.40)	< 0.001	1.30(1.19–1.43)	< 0.001
Categories								
< 3	Ref		Ref		Ref		Ref	
≥ 3	7.31(5.86–9.31)	< 0.001	4.39(3.47–5.55)	< 0.001	1.97 (1.52–2.56)	< 0.001	2.06(1.58–2.70)	< 0.001

Abbreviations: *OR* Odds ratio, *CI* Confidence interval

Model I: Adjusted for age and sex

Model II: Adjust for variables that, when added to this model, changed in effect estimate of more than 10%, included the covariates in Model I plus stroke, CCI, time of risk, central venous catheter, enteral tube feeding, Barthel Index, and Morse Fall Scale

Model III: Adjust for all of these variables, included the covariates in Model II plus adjusted for drinking status, smoking status, COPD, swallow disability, diabetes mellitus, peptic ulcer disease, moderate or severe renal disease, liver disease, congestive heart failure, solid tumor, admission category, indwelling urinary catheter, surgery, parenteral nutrition, other nosocomial infections, season of admission, antacids, sedatives, NSAID, systemic steroid, Inhaled steroid, and anticoagulant

Note: a total score of≥3 indicated a patient is “at nutritional risk”

After additional adjustment for stroke, CCI, time of risk, central venous catheter, enteral tube feeding, Barthel Index, and Morse Fall Scale (Model II), the association did not change (OR = 1.97;95% CI: 1.52, 2.56). Finally, in the fully adjusted model (Model III), the OR was 2.06 (95% CI: 1.58, 2.70). Similarly, per 1-point increase in the NRS score was associated with a 105,74,28, and 30% higher risk of NV-HAP in different multivariate logistic regression models.

### Sensitivity and subgroup analyses

A total of 174 patients with missing NRS score data were found. After excluding 40 patients with repeated admissions, 134 patients with missing NRS score data were retained, accounting for 0.2% of all analysed patients (134/ 67,280). There was no significant difference in most baseline characteristics, including the Charlson comorbidity index, between the two groups (Table S3 in the Supplementary Appendix).

In the sensitivity analyses, similar results were observed in analyses using a GEE method with a logit link and exchangeable correlation matrix (Table S4 in the Supplementary Appendix).

**Fig. 3:**
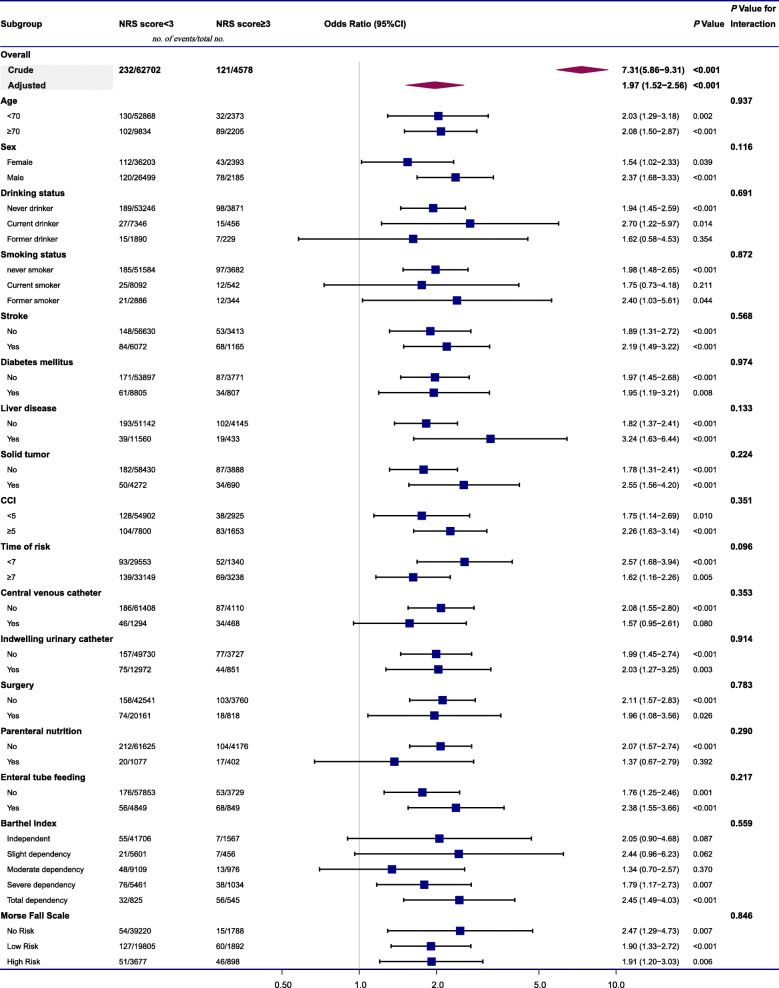
The association between NRS score (≥3/< 3) and the risk of NV-HAP in various subgroups. Values were adjusted for age, sex, stroke, CCI, time of risk, central venous catheter, enteral tube feeding, Barthel Index, and Morse Fall Scale. Abbreviations: NRS, nutritional risk screening; NV-HAP, nonventilator hospital-acquired pneumonia; CCI, charlson comorbidity index; CI, confidence interval. Note: The *p* value for interaction represents the likelihood of interaction between the variable and the NRS score

We further performed stratified and interaction analyses to assess the effect of the NRS score (< 3/≥3) on NV-HAP in various subgroups (Fig. 3). In the subgroup analyses, the NRS score was associated with a greater risk of NV-HAP in most subgroups. The difference was not statistically significant in some subgroups, probably due to the limited sample size. Tests for interaction showed no statistically significant differences for effect modification by other covariates (all *P*value for interaction > 0.05).

## Discussion

Given the alarming increasing burden of NV-HAP, there is an urgent need to accurately identify high-risk patients with NV-HAP. To our knowledge, this is the first study to demonstrate the usefulness of NRS score in predicting NV-HAP. The main finding of this study is that NRS score is independently associated with the development of NV-HAP. This association was also robust in different regression models and different subgroups. Furthermore, no significant interactive effects of the NRS score and NV-HAP were found, suggesting that the association between the NRS score and NV-HAP is consistent and stable, regardless of the patient’s characteristics. These findings suggest that the NRS score may assist risk stratification, to identify specific subgroups of patients at a higher risk of developing NV-HAP. In addition, these results provide ideas for developing strategies to reduce the incidence of NV-HAP based on nutritional.

There is no clear and unified criteria for the diagnosis of malnutrition [28]. Over the years, several screening tools have been developed to identify malnutrition risk [29, 30], such as the NRS-2002, the Malnutrition Universal Screening Tool (MUST), Mini Nutritional Assessment (MNA), and Subjective Global Assessment (SGA). The NRS-2002 has been tested and used in hundreds of randomized controlled trials [15] and was found to be an accurate and reliable screening tool if applied by trained staff [30]. In contrast to SGA and MNA, NRS 2002 takes much less time to perform and requires less rigorous examiner training [31, 32]. In addition, the NRS-2002 can more accurately identify individuals at high nutritional risk or have poor nutritional status compared to MUST [31]. For these reasons, our hospital chose the NRS-2002 as the screening tool.

Malnutrition is a significant issue closely related to infection as it can impair normal immune system development and cause severe damages to mucosal epithelial barriers in the mucosal tissues [33]. Several studies have linked nutritional parameters with the development and progression of pneumonia. A recent study reported that age and early postoperative hypoalbuminemia were independent risk factors for postoperative pneumonia in patients undergoing hip fracture surgery [34]. Another study revealed that in patients under the age of 65, age, serum cholinesterase and total cholesterol levels were associated with both the severity of pneumococcal pneumonia and length of hospital stay [35]. Besides, several studies have shown that nutritional risk scores using these tools associated with HAIs. A recent cross-sectional study observed a strong positive association between the MUST score and the prevalence of HAIs [12]. Similar findings were obtained in a longitudinal study of hospitalized elderly patients by Gamaletsou et al. [36] Several studies have demonstrated the predictive value of preoperative nutrition risk in various surgical site infections [37–41]. Although the association between the nutrition risk and the risk of HAIs, especially in surgical site infection, has been reported, the role of the nutritional risk screening score in NV-HAP remains poorly undefined.

Scientific evidence about identifying modifiable risk factors or predictive factors of NV-HAP is meager and of limited quality. A case-control study of 132 patients showed that age, the use of antacids, and central nervous system disease were independent risk factors of NV-HAP, but the poor nutritional status was not [9]. However, this conclusion should be interpreted with caution due to the small sample size of the study. In a systematic review and meta-analysis that included 144 studies, Schreiber et al. assessed the proportion of HAIs prevented by multifaceted interventions and only two of the studies involved NV-HAP [42]. To the best of our knowledge, no study has assessed the effectiveness of nutritional control measures on a large scale of patients with NV-HAP. Only one study proposed a procedure of healthy control as a preventive measure of postoperative pneumonia.

Hiramatsu et al. [43] conducted a historical case-control study in which they examined the influence of a preoperative care bundle, including a procedure of nutritional control, three breathing exercise procedures, two oral care procedures, and smoking cessation, on the incidence of postoperative pneumonia among esophageal cancer patients. The results indicated that the risk of postoperative pneumonia was reduced by 84% (OR = 0.16; 95%CI: 0.01, 0.94) after implementation of the care bundle. However, the independent role of nutritional control procedure in the successful implementation of practice measures has not been clarified. Thus, understanding what works, why, and for whom is pivotal to the effective management of patients at risk of postoperative pneumonia [44]. The present findings provide a new theoretical perspective to the role of NRS score as a predictor of nonventilator hospital-acquired pneumonia.

The strengths of this study include inclusion of a large patient cohort, collection of detailed covariate data, adjusted patients’ covariates exposure time to the hospital environment, and implementation of sensitivity analysis to test the robustness of the results. The study also conformed to the recommended diagnostic criteria instead of the International Classification of Diseases codes to diagnose NV-HAP [10] as a critical stronghold. Also, misclassification bias was minimized as each case of NV-HAP was rigorously reviewed and co-confirmed by a clinician and a senior infection control practitioner.

Nonetheless, there are also some limitations that are worth mentioning. First, as with any observational study, we cannot rule out the possibility of residual confounders despite controlling for 31 variables in our models. Furthermore, the use of retrospective data may introduce selection biases. Therefore, in the sensitivity analyses, we tested consecutive patients by the GEE method to minimize selection bias. Secondly, there were likely possible NRS score measurement errors. Although the ward nursing staff were well-trained to conduct nutritional risk screening, there still could be measurement bias as the screening was carried out by different nurses. Finally, the numbers of patients in some subgroups were small, yielding limited statistical power as this may conceal some meaningful results in both stratified and interaction analyses.

In summary, this study shows that the NRS score is an independent predictor of NV-HAP, irrespective of the patient’s characteristics. NRS-2002 as a simple and rapid tool for nutritional risk screening, has the potential to be applied as a convenient tool for risk stratification of adult hospitalized patients with different NV-HAP risks.

## Supplementary Information

**Additional file 1 **:**Table S1.** Variance inflation factor test for covariates. **Table S2.**Associations of covariates with NV-HAP.**Table S3.** Differences in baseline characteristics between missing and non-missing NRS score data patients. **Table S4.** Association between nutritional risk screening score and NV-HAP in GEE regression model (*N* = 91,604).

## Data Availability

The datasets used and analysed during this study are available from the corresponding author on reasonable request.
