# Supplementary material for: Nutritional risk screening score as an independent predictor of nonventilator hospital-acquired pneumonia: a cohort study of 67,280 patients
Source: BMC Infect Dis. 2021 Apr 1;21:313. doi: 10.1186/s12879-021-06014-w (PMC8013169; doi:10.1186/s12879-021-06014-w)
Supplement: Supplementary file 1 — Additional file 1 :Table S1. Variance inflation factor test for covariates. Table S2.Associations of covariates with NV-HAP.Table S3. Differences in baseline characteristics between missing and non-missing NRS score data patients. Table S4. Association between nutritional risk screening score and NV-HAP in GEE regression model (N = 91,604). [file 12879_2021_6014_MOESM1_ESM.docx]

Supplementary Appendix

Zhihui Chen^1, 2^, MPH, Hongmei Wu^2^, RN, Jiehong Jiang^3^, BE, Kun Xu^3^, BE, Shengchun Gao ^2^, RN, Le Chen^2^, RN, Haihong Wang^2^, RN^,^ Xiuyang Li^1^, phD

^1^ Department of Epidemiology and Biostatistics, and Centre for Clinical Big Data Statistics, Second Affiliated Hospital, Zhejiang University College of Medicine, Hangzhou, China.

^2^Department of Infection Control, Wenzhou people's Hospital, Wenzhou, China.

^3^XingLin Information Technology Company, Hangzhou, China.

Corresponding author:

Xiuyang Li, phD

Department of Epidemiology and Biostatistics, and Centre for Clinical Big Data Statistics, Second Affiliated Hospital, Zhejiang University College of Medicine, 866 Yuhangtang Road, Hangzhou, 310058, China

E-mail: lixiuyang@zju.edu.cn

Tel: +86-571-8820-8192

**Table of Contents Page Page**

**Table S1. Variance inflation factor test for covariates 3-4**

**Table S2. Associations of covariates with NV-HAP 5-7**

**Table S3.** **Differences in baseline characteristics between 8-10**

**missing and non-missing NRS score data patients**

**Table S4. Association between nutritional risk screening score 11**

**and NV-HAP in GEE regression model(N=91604)**

**Table S1. Variance inflation factor test for covariates**

| Various | VIF |
| --- | --- |
| Age | 5 |
| sex | 1.5 |
| drinking status | 1.4 |
| Smoking status | 1.6 |
| COPD | 1.2 |
| Swallow disability | 1 |
| Stroke | 1.5 |
| Diabetes Mellitus | 1.5 |
| Peptic ulcer disease | 1.1 |
| Moderate or severe renal disease | 1.4 |
| Liver Disease | 1.4 |
| Congestive heart failure | 1 |
| Solid tumor | 2 |
| CCI | 9.8 |
| Time of risk | 1.3 |
| Admission category | 1.4 |
| Central venous catheter | 1.3 |
| Indwelling urinary catheter | 1.8 |
| Surgery | 1.9 |
| Parenteral nutrition | 1.2 |
| Enteral tube feeding | 1.1 |
| Barthel Index | 1.5 |
| Morse Fall Scale | 1.9 |
| Other nosocomial infections | 1.1 |
| Season of admission | 1 |
| Use of antacids | 1.2 |
| Use of sedatives | 1.1 |
| Use of NSAID | 1.1 |
| Use of Steroid, systemic | 1.1 |
| Use of Steroid, inhaled | 1.1 |
| Use of anticoagulant | 1.2 |
| NRS score | 1.1 |

Abbreviations: VIF, variance inflation factor; COPD, chronic obstructive pulmonary disease; CCI, charlson comorbidity index; NSAID, nonsteroidal anti-inflammatory drug; NRS, nutritional risk screening.

A VIF greater than 10 was considered statistically significant for collinearity.

**Table S2. Associations of covariates with NV-HAP**

|  | logistic regression model | | |  | GEE model | | |
| --- | --- | --- | --- | --- | --- | --- | --- |
| Covariates | Basic model |  | Complete model |  | Basic model |  | Complete model |
| Initial regression coefficient | 1.99 |  | 0.72 |  | 1.80 |  | 0.71 |
| Age | - |  | - |  | - |  | - |
| Male | - |  | - |  | - |  | - |
| drinking status | 1.97 |  | 0.73 |  | 1.80 |  | 0.70 |
| Never drinker |  |  |  |  |  |  |  |
| Current drinker |  |  |  |  |  |  |  |
| Former drinker |  |  |  |  |  |  |  |
| Smoking status | 1.96 |  | 0.73 |  | 1.79 |  | 0.71 |
| Never smoker |  |  |  |  |  |  |  |
| Current smoker |  |  |  |  |  |  |  |
| Former smoker |  |  |  |  |  |  |  |
| COPD | 1.98 |  | 0.72 |  | 1.80 |  | 0.71 |
| Swallow disability | 1.96 |  | 0.72 |  | 1.79 |  | 0.71 |
| Stroke | 1.64^*^ |  | 0.72 |  | 1.60^*^ |  | 0.71 |
| Diabetes Mellitus | 1.96 |  | 0.72 |  | 1.82 |  | 0.70 |
| Peptic ulcer disease | 1.98 |  | 0.73 |  | 1.80 |  | 0.71 |
| Moderate or severe renal disease | 1.98 |  | 0.72 |  | 1.78 |  | 0.72 |
| Liver disease | 1.99 |  | 0.73 |  | 1.80 |  | 0.72 |
| Congestive heart failure | 1.96 |  | 0.71 |  | 1.78 |  | 0.70 |
| Solid tumor | 1.85 |  | 0.72 |  | 1.60^*^ |  | 0.71 |
| CCI | 1.39^*^ |  | 0.75 |  | 1.31^*^ |  | 0.74 |
| Time of risk | 1.98 |  | 0.61^*^ |  | 2.15^*^ |  | 0.63^*^ |
| Admission category | 1.84 |  | 0.73 |  | 1.73 |  | 0.70 |
| Internal medicine |  |  |  |  |  |  |  |
| Surgery |  |  |  |  |  |  |  |
| gynaecology |  |  |  |  |  |  |  |
| Emergency department |  |  |  |  |  |  |  |
| ICU |  |  |  |  |  |  |  |
| Central venous catheter | 1.66^*^ |  | 0.75 |  | 1.55^*^ |  | 0.79^*^ |
| Indwelling urinary catheter | 2.01 |  | 0.73 |  | 1.79 |  | 0.70 |
| Surgery | 1.98 |  | 0.69 |  | 1.80 |  | 0.69 |
| Parenteral nutrition | 1.87 |  | 0.71 |  | 1.67 |  | 0.71 |
| Enteral tube feeding | 1.74^*^ |  | 0.78 |  | 1.61^*^ |  | 0.79^*^ |
| Barthel Index | 1.06^*^ |  | 1.02^*^ |  | 1.03^*^ |  | 0.98^*^ |
| Independent |  |  |  |  |  |  |  |
| Slight dependency |  |  |  |  |  |  |  |
| Moderate dependency |  |  |  |  |  |  |  |
| Severe dependency |  |  |  |  |  |  |  |
| Total dependency |  |  |  |  |  |  |  |
| Morse Fall Scale | 1.56^*^ |  | 0.73 |  | 1.52^*^ |  | 0.71 |
| No Risk |  |  |  |  |  |  |  |
| Low Risk |  |  |  |  |  |  |  |
| High Risk |  |  |  |  |  |  |  |
| Other nosocomial infections | 1.97 |  | 0.73 |  | 1.80 |  | 0.71 |
| Season of admission | 1.98 |  | 0.73 |  | 1.81 |  | 0.70 |
| Spring |  |  |  |  |  |  |  |
| Summer |  |  |  |  |  |  |  |
| Fall |  |  |  |  |  |  |  |
| Winter |  |  |  |  |  |  |  |
| Use of antacids | 1.92 |  | 0.72 |  | 1.71 |  | 0.71 |
| Use of sedatives | 1.92 |  | 0.73 |  | 1.75 |  | 0.71 |
| Use of NSAID | 1.91 |  | 0.71 |  | 1.71 |  | 0.70 |
| Use of Steroid, systemic | 1.99 |  | 0.73 |  | 1.80 |  | 0.70 |
| Use of Steroid, inhaled | 1.89 |  | 0.74 |  | 1.71 |  | 0.70 |
| Use of anticoagulant | 1.95 |  | 0.71 |  | 1.71 |  | 0.70 |

Abbreviations: GEE, generalized estimation equation; NV-HAP: Nonventilator hospital-acquired pneumonia; COPD, chronic obstructive pulmonary disease; CCI, charlson comorbidity index; ICU, intensive care unit; NSAID, nonsteroidal anti-inflammatory drug.

Adjust for variables that, when added in the basic model or removed in the complete model, changed in effect estimate of more than 10%.

* indicates a change of more than 10% compared with the initial regression coefficient.

**Table S3.** **Differences in baseline characteristics between** **missing and** **non-missing NRS score data patients**

| Demographics | non-missing data patients  (n=67280) | Missing data patients  (n=134) | *P* value |
| --- | --- | --- | --- |
| Age(years), median (Q1-Q3) | 51 (37-65) | 56 (33-66) | 0.465 |
| Male, n (%) | 28684 (42.6) | 62 (46.3) | 0.395 |
| Drinking status, n (%) |  |  | 0.118 |
| Never drinker | 57117 (84.9) | 109 (81.3) |  |
| Current drinker | 7802 (11.6) | 16(11.9) |  |
| Former drinker | 2119 (3.1) | 9 (6.8) |  |
| Missing | 242(0.4) | 0 (0.0) |  |
| Smoking status, n (%) |  |  | 0.546 |
| never smoker | 55266 (82.1) | 106 (79.1) |  |
| Current smoker | 8634 (12.9) | 18 (13.5) |  |
| Former smoker | 3230 (4.8) | 9 (6.7) |  |
| Missing | 150(0.2) | 1 (0.7) |  |
| Comorbidities, n (%) |  |  |  |
| COPD | 803 (1.2) | 4 (3.0) | 0.057 |
| Swallow disability | 126 (0.2) | 0 (0.0) | 0.616 |
| Stroke | 7237 (10.8) | 22 (16.4) | 0.035 |
| Diabetes mellitus | 9612 (14.3) | 17 (13.0) | 0.669 |
| Peptic ulcer disease | 2236 (3.3) | 4 (3.0) | 0.827 |
| Moderate or severe renal disease | 2944 (4.4) | 5(3.7) | 0.716 |
| Liver disease | 11993 (17.8) | 16 (11.9) | 0.075 |
| Congestive heart failure | 328 (0.5) | 0 (0.0) | 0.418 |
| Solid tumour | 4962 (7.4) | 7 (5.2) | 0.341 |
| CCI (points), median (Q1- Q3) | 1 (0-3) | 1 (0-4) | 0.736 |
| Time of risk(days), median (Q1- Q3) | 7 (4-10) | 7 (3-10) | 0.628 |
| Admission category, n (%) |  |  | <0.001 |
| Internal medicine | 27769 (41.3) | 48 (35.8) |  |
| Surgery | 19556 (29.1) | 32 (23.9) |  |
| [Gynaecology](http://www.baidu.com/link?url=_rGEFXM1wfYLtaxPtIAHhsdchHBATytzNZV1Sgfb_CBxcx-HXt7y0Ehn0uh-_v8EyDzw7_ujEYpoaINlW4mf0rqP0zUTuuis5OzG79rtqmmafnkSXYmyGV0ASXgNpweRMaVppA-QFYn6n0PMfgKFuq) | 15548 (23.1) | 9 (6.7) |  |
| Emergency department | 3172 (4.7) | 21 (15.7) |  |
| ICU | 203 (0.3) | 9 (6.7) |  |
| Others | 1032 (1.5) | 15 (11.2) |  |
| Clinical procedure, n (%) |  |  |  |
| Central venous catheter | 1762 (2.6) | 6 (4.5) | 0.179 |
| Indwelling urinary catheter | 13823 (20.5) | 24 (17.9) | 0.451 |
| Surgery | 20979 (31.2) | 33 (24.6) | 0.102 |
| Parenteral nutrition | 1479 (2.2) | 11 (8.2) | <0.001 |
| Enteral tube feeding | 5698 (8.5) | 7 (5.2) | 0.178 |
| Other nosocomial infections, n (%) | 1137 (1.7) | 3 (2.2) | 0.623 |
| Season of admission, n (%) |  |  | 0.512 |
| Spring | 16431 (24.4) | 40 (29.9) |  |
| Summer | 18652 (27.7) | 35 (26.1) |  |
| Fall | 14497 (21.5) | 25 (18.7) |  |
| Winter | 17700 (26.3) | 34 (25.3) |  |
| In-hospital medications, n (%) |  |  |  |
| Antacids | 37406 (55.6) | 51 (38.1) | <0.001 |
| Sedatives | 6615 (9.8) | 12 (9.0) | 0.733 |
| NSAID | 6503 (9.7) | 8 (6.0) | 0.148 |
| Systemic steroid | 13231 (19.7) | 9 (6.7) | <0.001 |
| Inhaled steroid | 4281 (6.4) | 12 (9.0) | 0.220 |
| Anticoagulant | 8972 (13.3) | 24 (17.9) | 0.120 |
| NV-HAP | 353(0.5) | 2 (3.0) | 0.122 |

Abbreviations:NV-HAP: Nonventilator hospital-acquired pneumonia; COPD, chronic obstructive pulmonary disease; CCI, charlson comorbidity index; ICU, intensive care unit; NSAID, nonsteroidal anti-inflammatory drug.

**Table S4. Association between nutritional risk screening score and NV-HAP in GEE regression model(N=91604)**

| NRS score | Non-adjusted Model | |  | Model Ⅰ | |  | Model Ⅱ | |  | Model III | |
| --- | --- | --- | --- | --- | --- | --- | --- | --- | --- | --- | --- |
|  | OR (95% CI) | *P* value |  | OR (95% CI) | *P* value |  | OR (95% CI) | *P* value |  | OR (95% CI) | *P* value |
| Continuous, per 1-point increment | 1.75(1.67-1.85) | <0.001 |  | 1.57(1.49-1.65) | <0.001 |  | 1.22 (1.15, 1.29) | <0.001 |  | 1.24(1.17-1.32) | <0.001 |
| Categories |  |  |  |  |  |  |  |  |  |  |  |
| <3 | Ref |  |  | Ref |  |  | Ref |  |  | Ref |  |
| ≥3 | 6.04(4.90-7.44) | <0.001 |  | 4.14(3.46-4.96) | <0.001 |  | 1.93 (1.59, 2.33) | <0.001 |  | 2.03(1.66-2.47) | <0.001 |

Abbreviations: GEE, generalized estimation equation; NRS, nutritional risk screening.

Model I: Adjusted for age and sex.

Model II: Adjust for variables that, when added to this model, changed in effect estimate of more than 10%, included the covariates in Model I plus stroke, solid tumor, Charlson comorbidity index, time of risk, central venous catheter, enteral tube feeding, Barthel Index, Morse Fall Scale.

Model III: Adjust for all of these variables, included the covariates in Model II plus adjusted for drinking status, smoking statoscope, swallow disability, diabetes mellitus, peptic ulcer disease, moderate or severe renal disease, liver disease, congestive heart failure, admission category, indwelling urinary catheter, surgery, parenteral nutrition, other nosocomial infections, season of admission, antacids, sedatives, NSAID, systemic steroid, Inhaled steroid, and anticoagulant.

Note: a total score of≥3 indicated a patient is “at nutritional risk”.
